# Complexation of a Polypeptide-Polyelectrolytes Bioparticle as a Biomaterial of Antibacterial Activity

**DOI:** 10.3390/pharmaceutics14122746

**Published:** 2022-12-08

**Authors:** Carlos A. B. Ramirez, Mateus M. Carriero, Fernanda S. C. Leomil, Ricardo L. Moro de Sousa, Antonio de Miranda, Omar Mertins, Patrick D. Mathews

**Affiliations:** 1 Department of Biophysics, Paulista Medical School, Federal University of Sao Paulo (UNIFESP), Sao Paulo 04023-062, Brazil; 2 Department of Veterinary Medicine, University of Sao Paulo (USP), Pirassununga 13635-900, Brazil

**Keywords:** polypeptide antibacterial activity, *Acustisoma longipes* hemolymph, *Aeromonas dhakensis*, chitosan, alginate, giant vesicle electroporation

## Abstract

The development of biomaterials to enable application of antimicrobial peptides represents a strategy of high and current interest. In this study, a bioparticle was produced by the complexation between an antimicrobial polypeptide and the biocompatible and biodegradable polysaccharides chitosan-*N*-arginine and alginate, giving rise to a colloidal polyelectrolytic complex of pH-responsive properties. The inclusion of the polypeptide in the bioparticle structure largely increases the binding sites of complexation during the bioparticles production, leading to its effective incorporation. After lyophilization, detailed evaluation of colloidal structure of redispersed bioparticles evidenced nano or microparticles with size, polydispersity and zeta potential dependent on pH and ionic strength, and the dependence was not withdrawn with the polypeptide inclusion. Significant increase of pore edge tension in giant vesicles evidenced effective interaction of the polypeptide-bioparticle with lipid model membrane. Antibacterial activity against *Aeromonas dhakensis* was effective at 0.1% and equal for the isolated polypeptide and the same complexed in bioparticle, which opens perspectives to the composite material as an applicable antibacterial system.

## 1. Introduction

Bacterial infections and antibiotic resistance are among the main concerns in human and veterinary medicine. In the development of new strategies in the fight against bacterial threats, the potential of diverse drug delivery systems, such as micro and nanoparticles, liposomes, hydrogels, nanofibers, as carriers of antibacterial drugs and antimicrobial bioactives, has been evidenced [1,2,3,4,5,6,7,8]. Several natural polypeptides have been described as having efficient antibacterial activity, and they represent a broad-spectrum natural alternative against bacteria, which encourages increasing development for their effective application [9].

Polypeptides are generally cationic and amphiphilic, capable of antibacterial activity by interacting with the pathogen’s negatively charged components or cell surface receptors [10]. Most cationic antimicrobial peptides have amino acid residues within their sequences, which gives the possibility, by chemical modification, of designing new improved peptides with bacteriostatic and bactericidal activities. For instance, the peptide pexiganan, a synthetic analogue of magainin II, with 22 amino acids this peptide acts against infections of diabetic foot ulcers, inhibiting the formation of biofilms and bacterial adhesion, acting together and thus decreasing the minimum inhibitory concentration of the antibiotic imipenem [11].

The association of bioactives with micro or nanoparticles has proven advantages for allowing further specific applications, specially concerning pharmacological improvement concomitant with elimination or lowering of side effects, besides reduction of prolonged treatment regimens and costs. In this context, antimicrobial peptides were complexed with nanoparticles and the improved biological activity has been demonstrated [12,13,14,15]. Nanoparticles produced with biodegradable polymers have additionally proven promising application perspectives due to their higher biocompatibility and degradation in a biological milieu, providing reduced toxicity and safer administration of peptide-based drugs [16,17]. An important desired feature in application of antibiotics is boosting the antibacterial action by the promotion of targeted delivery provided by the micro or nanoparticles [18,19]. Accordingly, vapreotide carried in chitosan nanoparticles has shown superior in vitro antibacterial activity, if compared to the free peptide, against *Escherichia coli* and *Staphylococcus aureus* [20].

The chitosan biopolymer has a macromolecular structure, with bioactive and versatile physicochemical qualities of high interest to research and industrial biotechnology [21,22]. Additionally, alginate biopolymer has also been studied as a component in the administration of bioactive agents, such as drugs and proteins, acting in the controlled release of these molecules [23,24].

Previously, chitosan was modified by chemically linking arginine to part of the biopolymer monomers [25]. Arginine has the advantage of being positively charged in the biological media due to the guanidinium group (pKa 13). Thus, chitosan-*N*-arginine was complexed with the negatively charged alginate in the production of nanoparticles of pH-responsive structure, profitable for drug delivery via oral administration [26,27,28].

In the present study, a polypeptide was complexed with chitosan-*N*-arginine and alginate, providing a bioparticle intended for antibacterial applications. The bioparticle was obtained with the inclusion of JM72, which is a polypeptide produced from the isolated antibacterial peptide longipinene, extracted from the hemolymph of *Acustisoma longipes*, with molecular structure rich in arginine (R) and tryptophan (W) and with primary sequence: Ac-SGWLPGREWVWRWRGRVF-NH_2_ [29]. The JM72 polypeptide may be susceptible to degradation in the biological environment of the application pathway, resulting in no antibacterial activity. Therefore, the polypeptide carried in bioparticles can be a promising strategy to enable the application of JM72, avoiding its degradation during administration and at the same time optimizing its performance at the desired site of action.

The bioparticle as a result of complexation between the biopolymers and the polypeptide was thoroughly studied since its formation in controlled conditions by evaluating the thermodynamics of molecular interactions. As a polyelectrolytes bioparticle, which may be highly susceptible to the ionic effects of the application media [30,31], leading to structural variations that affect biological performance, the resulting bioparticle structure was further analyzed in low and high ionic strength solutions of different pH. Moreover, the new JM72-bioparticle interaction with lipid membrane of giant vesicles was evaluated in terms of pore edge tension and finally, the in vitro antibacterial activity was tested against *Aeromonas dhakensis*, a pathogenic bacteria found in fish and water streams and an increasingly recognized human pathogen [32,33,34].

## 2. Materials and Methods

### 2.1. Materials

The chitosan used (ChitoClear 43000, Primex, Siglufjordur, Iceland) has 95% deacetylation degree, average molecular weight Mw = 130 kDa (corresponding to an average of 797 monomers per molecule) and low viscosity (<20 cP). Sodium alginate (extracted from the brown algae *Macrocystis pyrifera*; Sigma-Aldrich, St. Louis, MO, USA) has 61% mannuronic acid and 39% guluronic acid, with average molecular weight Mw = 200 kDa (1143 uronic acid units) and low viscosity (5 to 40 cP). *L*-arginine (98%) was purchased from Sigma-Aldrich. JM72 polypeptide (extracted from *Acustisoma longipes* hemolymph) has a mean molecular weight of 2372 Da and was obtained and characterized as previously described [29]. Chitosan-*N*-arginine was obtained and characterized in our previous studies [25] and has 3.5% of chitosan monomers linked to arginine. All further reagents were of analytical grade and water was purified from MilliQ filter system (Millipore Corp., Burlington, VT, USA) to a total organic carbon value of less than 15 ppb and resistivity of 18 MΩ.cm.

### 2.2. Preparation of Bioparticles

Two 50 mL biopolymer solutions were prepared by shaking in acetate buffer (80 mM, pH 4.50 ± 0.02) the following concentrations: 0.1 mg/mL alginate solution and 1 mg/mL chitosan-*N*-arginine, both stirred at 2500 rpm for 3 min (Gehaka Vortex mixer) and then submitted for 30 min in an ultrasonic bath (40 kHz; 25 °C; Eco-Sonics Q3.0L, Brazil). Subsequently, the solutions were filtered with 0.8 μm filters (Millipore) to eliminate impurities. Finally, the alginate solution was titrated dropwise (2 mL/min) into the chitosan-*N*-arginine solution under magnetic stirring (1200 rpm; 22 ± 2 °C) and after the mixture was kept under stirring overnight, thus obtaining the colloidal bioparticles through electrostatic interactions between the two oppose charged macromolecules [25]. For preparation of particles with the antibacterial polypeptide JM72, the same was added at different concentrations 2.5, 7.5 and 15 μM, in chitosan-*N*-arginine solution, before the addition of alginate, leaving it under stirring overnight (1200 rpm; 22 ± 2 °C). After preparation, the colloidal particles were frozen with dry ice and lyophilized for 48 h, obtaining them as a fine dry powder. The material was kept in a refrigerator at 4 °C.

### 2.3. Preparation of Giant Vesicles and Electroporation

For the biological model membrane, giant unilamellar vesicles (GUVs) were produced with POPC (1-palmitoyl-2-oleoyl-*sn*-glycero-3-phosphocholine; 99%, Avanti Polar Lipids) dissolved in chloroform (1 mg/mL) and using the electroformation procedure [35]. Briefly, the lipid film for hydration was made using a 10 µL glass micro syringe previously washed with chloroform and through which 30 µL of lipid solution were transferred over two glass slides with an indium tin oxide surface, spreading in a way to produce an apparently uniform thin film. The lipid films over the two slides were completely dried with nitrogen gas stream and by keeping in a desiccator under vacuum for 2 h. The slides were superimposed so that the film of each one was delimited and spaced by a Teflon spacer with a thin layer of grease that allows for keeping the assembled chamber closed during the electroforming process. Then, 3 mL of sucrose solution (2 mM) were slowly injected through a small hole in the spacer, avoiding the formation of air bubbles, until the chamber was completely filled. The seal was kept secure with metal clamps, preventing leaking, and one end of each glass slide was connected with electrodes from an electrical generator (Gratten ATF20B, 20 MHz) programmed with the following specifications: 10 Hz frequency; amplitude 1.5–1.5 Vpp; 50 Ω output; for 2 h. After formation, the obtained GUVs suspension was carefully transferred to an Eppendorf vial.

For the observation and electroporation experiments, 100 µL of GUVs suspension was placed in an electroporation chamber (Eppendorf, Hamburg, Germany), consisting in two parallel platinum electrodes 500 µm apart. Subsequently, 870 µL of glucose solution (1.9 mM) with 130 µL of NaCl solution (0.5 mM) and suspension of JM72-bioparticles (15 µM JM72) dispersed in the same glucose solution at amounts of 10, 20 and 30 µL for independent experiments were carefully included in the cell with micropipette. GUVs were observed under an inverted microscope (Zeiss, Axiovert 200, Jena, Germany) equipped with a 20×, NA 0.4 objective and a sCMOS PCO.edge 4.2 digital camera. The employed acquisition rate was between 400 and 600 frames per second (fps). The chamber was connected to a multiporator (Eppendorf 940000602 Multiporator^®^) for the application of electrical pulses (3 kV/cm, 150 µs). From the pulse application and subsequent opened pores, individual pore dynamics was analyzed. The theoretical work from Brochard-Wyart [36] describes pore dynamics in a spherical vesicle of radius *R* as a four-stage process: (1) growth, (2) stabilization, where it reaches its maximum radius, (3) leakage stage, which is the longest, and finally (4) quick closing. The third stage, which is linear, can be used for pore edge tension determination. By recording the time dependence of the pore closure, pore edge tension (γ) can be assessed by means of Equation (1):

(1) $$R^{2}ln\left(r\right)=-\frac{2\gamma }{3\pi \eta }t+C$$

where *R* is the GUV radius after pore closure, *r* is the pore radius over time *t*, *η* is the viscosity of the aqueous solution and *C* is a constant dependent on each experiment. Upon pulse application, pore dynamics was evaluated automatically with PoET [37], where for *η* we used 1.133 × 10^−3^ Pa.s. The mean values of edge tension for independent groups were compared via monofactorial analysis of variance followed by Bonferroni post-test.

### 2.4. Dynamic Light Scattering and Zeta Potential

The hydrodynamic diameters and surface charge of bioparticles not containing and containing the polypeptide JM72 (15 µM) were characterized in buffers with pH 2.5 (citrate), 4.5 (acetate), 6.5 (phosphate) and 7.8 (phosphate), all in two concentrations of 8 mM and 40 mM. Quasi-elastic light scattering and zeta potential measurements were performed with a Nano-ZS90 Malvern ZetaSizer equipment (Malvern Instruments, UK) operating with a 4 mW HeNe laser at a wavelength of 632.8 nm in a temperature-controlled chamber at 25 °C and detection at an angle of 173°. 0.1 mg/mL of bioparticle was dispersed in each buffer. Then, measurements were carried out in the equipment folded capillary cell with data acquisition and analysis using Malvern software. The intensity-weighted size distribution was obtained by fitting data with a discrete Laplace inversion routine. Size determination was made using the Stokes–Einstein relation and the polydispersity was acquired by cumulant analysis of the obtained correlation functions applying the amplitude of the correlation function and the relaxation frequency. The second-order cumulant was used to compute the polydispersity of each sample. Each result corresponds the analysis of three consecutive measurements.

Zeta potential based on laser Doppler velocimetry was concomitantly measured with data acquired performing at least 100 runs per sample. The electrophoretic mobility was converted to zeta potential in mV using the Helmholtz Smoluchowski relationship.

### 2.5. Isothermal Titration Calorimetry (ITC)

ITC measurements were performed in a MicroCal Inc. (Northampton, MA, USA) microcalorimeter. The equipment cell, which has a volume of 1.442 mL, was filled with 0.5 mM chitosan-*N*-arginine solution in 80 mM acetate buffer, pH 4.50, without JM72 polypeptide or containing 2.5, 7.5 and 15 µM of the polypeptide, respectively, for individual experiments. On the other hand, alginate solution (5 mM) in the same acetate buffer was filled in the injection syringe to dose 1 aliquot of 2 µL, followed by 27 aliquots of 10 µL of this solution into the cell, at 400 s intervals, mixing at 307 rpm at 25 °C. The data acquisition and analyses were carried out with Origin software provided by MicroCal and the data fitting was performed applying the single set of identical binding sites model, as previously described [38].

### 2.6. In Vitro Antibacterial Analysis

The *Aeromonas dhakensis* bacteria was isolated from a moribund juvenile pacu fish (*P. mesopotamicus*) obtained from the fish farm of the National Center for Continental Fish Research and Conservation (CEPTA/ICMBio) in the municipality of Pirassununga, State of Sao Paulo, Brazil and was identified by phenotypic and molecular identification, as described in details in Carriero et al. [39]. The colonies were inoculated in 1 mL of trypticase soy broth (TSB; BD Difco^TM^) and incubated for 24 h at 30 °C. The inhibitory effects of the free polypeptide JM72 and the same carried in the bioparticles was accessed by means of turbidity measurements. Optical densities (OD) of bacterial cultures were measured at 590 nm and adjusted to 1.5. For antibacterial analysis, aliquots of JM72 or JM72-bioparticle suspensions (15 µM JM72) in acetate buffer (pH 5.5) and autoclaved at 121 °C for 20 min were added to the bacterial broth and the bacteria proliferation assays were conducted by measuring the turbidity of *Aeromonas dhakensis* incubated in the samples from 0 to 0.8% (*w*/*v*) in 96-well microtiter plates. The kinetics of bacterial growth was measured at 30 °C during 24 h and keeping constant slight shaking, in a multiskan microplate reader (Thermo Fisher Scientific Inc., Waltham, MA, USA). Triplicate of each condition was performed and data expressed as mean ± standard error of mean.

## 3. Results and Discussion

### 3.1. Thermodynamics of Interaction in the Bioparticles Production

To evaluate the physicochemical interaction between the polyelectrolytes during the bioparticle formation process, the ITC technique was used, which provided the heat released in the titration of ionized alginate (Alg) in protonated chitosan-*N*-arginine (CHarg) and JM72 polypeptide.

**Figure 1:**
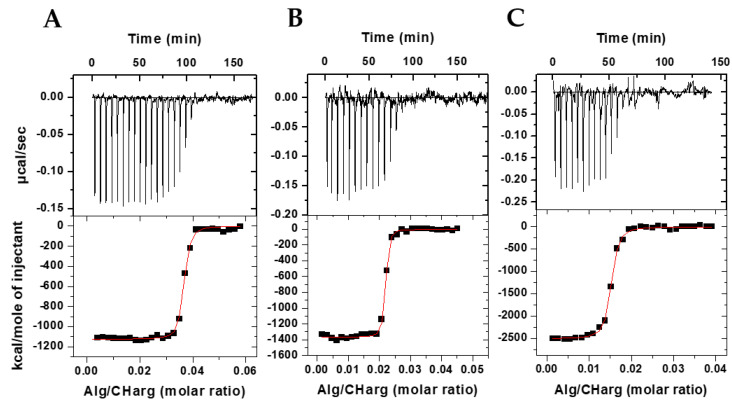
ITC results at 25 °C, pH 4.5, for interaction between alginate and CHarg in the presence of JM72 polypeptide at different concentrations: (**A**) 2.5 µM; (**B**) 7.5 µM; (**C**) 15 µM.

Figure 1 shows in the upper panels the heat peaks released with each addition of alginate solution to CHarg and JM72 solution. It is evident that negative enthalpy variation occurs in an exothermic process that gradually decreases in intensity with each addition, until when there are no more interaction sites available between the oppositely charged polyelectrolytes.

In the lower panels are shown the integrated heats resulting from the interaction as a function of the Alg/CHarg molar ratio and which present sigmoidal behavior, evidencing, from a certain molar ratio, the neutralization of protonated chitosan with negatively charged alginate. The sigmoid curve is a result of a nonlinear least squares fit considering a 1:1 interaction between alginate monomer and CHarg monomer providing the equilibrium constant, *K*, the number of binding sites, *N*, and the enthalpy variation, ΔH, of the complexation of macromolecules during particle formation. With *K*, we calculate the Gibbs energy applying ΔG = −RTln(55.55 *K*), where ΔG is defined for standard state of molar fraction, T is the temperature and 55.55 introduces the water concentration. Applying TΔS = ΔH − ΔG the entropy gain is also estimated. Thus, a complete thermodynamic characterization of the particle production was provided (Table 1).

The number of binding sites, *N*, representing the number of ionized alginate monomers interacting with protonated CHarg monomers in the saturation of binding sites, was 0.63 for particles in absence of JM72 polypeptide (Table 1), in accordance with a previous study in the same conditions [25]. This represents 1/*N* ~ 1.6 CHarg monomers per alginate monomer at saturation. However, with inclusion of JM72 in the system, this proportion changes significantly. For the 2.5 µM concentration, the 1/*N* ratio was 27; for the 7.5 µM concentration, the ratio was 44 and for 15 µM it was 68. This large increase in interaction sites with a JM72 concentration may be related to changes in molecular conformations of the polyelectrolytes in solution, as well as the positive charges added by the peptide, with its protonated amine groups and arginine. This increase in accessible interaction sites is, therefore, directly related to the increase in JM72 concentration, evidencing the contribution of the polypeptide charges in complexation and formation of the bioparticles.

Likewise, the equilibrium constant, *K*, resulted in higher values (Table 1), confirming that association between the polyelectrolytes is more favored with the concentrations of JM72, as well as the negative variation of enthalpy, ΔH, which had a substantial increase denoting strong exothermic interaction of greater intensity with increase in concentration of JM72. The greater and negative variation of free energy, ΔG, also evidences that formation of particles is more favored with JM72 and finally the negative variation of entropy, ΔS, which decreases significantly, shows that the polypeptide favors the association of components forming condensed structures and leading to a lower entropy state at a constant temperature. Overall, these results evidence that JM72 thermodynamically favored the production of bioparticles.

### 3.2. Colloidal Structure

The colloidal structure of polyelectrolytes particles may strongly depend on ionic strength of the solution where they are dispersed. Furthermore, the effective application performance of these particles is dependent on the level of protonation and ionization of chargeable groups of the macromolecules. Therefore, the structure of produced bioparticles was evaluated in relatively low and high ionic strength. Buffers were besides selected and applied with the aim of simulating acidity and alkalinity conditions, which relay the gastrointestinal tract.

Aiming to evaluate the contribution of the polypeptide on the structure of the bioparticle, the structural characterization of bioparticles without JM72 is presented. Considering the concentration of 8 mM buffer solutions (Table 2), at pH 2.5 the bioparticle had an average hydrodynamic diameter of 361 nm which, despite the relatively high polydispersity (0.52), showed a single size distribution population. From pH 4.5 onwards, there was partial formation of micro and sub-micrometric structures in different proportions, and especially at pH 4.5, high presence of microparticles was characterized. The zeta potential provided positive values for the bioparticles at all pHs with similar intensity at pH 2.5 and 4.5 and with a reasonable decrease at pH 6.5 and 7.8.

Considering the concentration of 40 mM buffer solutions (Table 3), at pH 2.5 the bioparticle had an average hydrodynamic diameter of 101 nm with relatively low polydispersity (0.21). However, in the other pHs, there was also an increase in sizes with the presence of microparticles, mainly at pH 4.5. The zeta potential showed results with greater dependence on pH, with positive values at pHs 2.5 and 4.5 and negative at pHs 6.5 and 7.8.

The change in conformation of polyelectrolytes in aqueous solutions largely depends on pH of a solution and its ionic strength. Thus, evaluation of the influence of ionic concentration becomes important to assess the stability of bioparticles in aqueous solution. For CHarg-Alg bioparticles, it should be considered that the pKa of chitosan is approximately 6. In addition, arginine chemically bound to chitosan in 3.5% of monomers has a pKa of the guanidinium group of approximately 13. In addition, alginate has a pKa of 3.4 for mannuronic groups and 3.7 for guluronic groups. These characteristics delimit the structural behavior of the two polyelectrolytes in solution. Considering chitosan-*N*-arginine, protonation of amino groups therefore occurs at acidic pHs 2.5 and 4.5, although the arginine groups also remain protonated at higher pHs, thus providing a certain amount of positive charge at pHs 6.5 and 7.8. Alginate, on the other hand, is not ionized at pH 2.5, but in the other pHs, where it provides negative charges to the bioparticles. Evidently, under conditions of a low concentration of the buffer solutions, the amount of dissociated acid available to protonate chitosan is lower than at a high concentration. Therefore, the different results, comparing Table 2 with Table 3, are related to the concentration of buffer solutions. At pH 2.5 the bioparticle showed a single population size in both buffer concentrations, but at the highest concentration (40 mM) the average size and polydispersity were much smaller, indicating that the bioparticle structure is more compact. This result shows that the higher ionic concentration better stabilized the particle in terms of size, although zeta potential showed a lower positive value in 40 mM buffer that may be related to the ionic layer near the surface of the bioparticle in solution, which must be larger and more dispersed at a concentration of 40 mM, leading to a lower zeta potential. From pH 4.5, the ionization of alginate significantly influences the structural characteristics, leading to partial aggregation and formation of microparticles. The presence of positive charges on chitosan and negative charges on alginate creates greater electrostatic attraction between bioparticles, which justifies aggregation. Although in the 40 mM solution of pH 4.5 zeta potential was higher, it was not enough to prevent the formation of microparticles, which resulted in expressive polydispersity.

The negative charges of ionized alginate provided a slightly negative zeta potential at pH 6.5 and 7.8 only in 40 mM buffer solutions. This indicates that at a low ionic concentration, the quantity of ions was not sufficient to provide inversion of surface charge of the bioparticles. The result may also indicate that at a low ionic concentration, chitosan predominates on the surface of the particle, keeping surface charge close to neutrality in this case, which may be related to its higher structural flexibility [40,41] and relatively lower molar mass.

Now, considering the bioparticles containing polypeptide JM72 (15 µM) in the structure, in buffers with a concentration of 8 mM (Table 4), at pHs 2.5, 4.5 and 6.5 varied results of hydrodynamic diameter and notably the presence of microparticles and relatively high polydispersity were evidenced. However, at pH 7.8, only a single population of particles with an average diameter of 203 nm was characterized, despite the significant polydispersity, showing that at alkaline pH there is compaction of the structure and absence of larger aggregates. On the other hand, zeta potential showed pH-dependent results with positive values at acidic pHs that decreased in intensity with increasing pH, reaching a slightly negative value at alkaline pH.

Considering the concentration of 40 mM buffer solutions (Table 5), different from the low ionic concentration solutions, at pH 2.5 the bioparticle with JM72 presented a single mean hydrodynamic diameter of 88 nm, indicating absence of aggregates. In the other pHs, the presence of microparticles was notably characterized at pH 4.5 and in a low relative proportion at pH 7.8. At pH 6.5, again only one population with a mean diameter of 334 nm was identified. Polydispersity was significantly high (0.86) at pH 4.5 with a high proportion of aggregates. On the other hand, zeta potential showed low dependence on pH, and the values varied close to neutrality, despite being slightly positive at acidic pHs and negative at alkaline pH.

For JM72-bioparticles, structural and charge contribution of the polypeptide must be considered, which has a high number of amino groups and also arginine, and is therefore similar to CHarg at these points. Comparing Table 4 with Table 5, it is clear that at pH 2.5 the low ionic concentration did not provide a favorable condition for complete protonation, which may have led to partial formation of aggregates. At pH 4.5, the presence of aggregates must have been generated again by ionization of alginate and occurred at low and high buffer concentrations and similarly at pH 6.5. At pH 7.8, where the amino groups are deprotonated, aggregates were present in the higher buffer concentration but absent at the lower concentration, which is an intriguing result. Possibly, the electrostatic interaction of the positive charges of arginine guanidine groups with the negative charges of alginate carboxylic groups was favored in the condition of a higher ionic concentration, but unfavored in the lower concentration.

Regarding zeta potential, the difference between the values characterized at pH 2.5 for the two conditions was remarkable. At a low ionic concentration, the mean value of 31.1 mV is significantly higher than the mean value of 2.4 mV for the high concentration of buffer. Adding to this, the results of having aggregates in the first case and of having a single structure with an average diameter of 88 nm in the second—the smallest characterized in the study—evidence the importance of ionic concentration of the medium as an influential parameter in the structure of the bioparticle.

It is also worth pointing out that the bioparticle without JM72 also presented a single and reduced hydrodynamic diameter, with an average value of 101 nm, only at pH 2.5 in the high buffer concentration condition (Table 3). These results indicate that citrate buffer at a high concentration of 40 mM is efficient for compacting the bioparticles. Additionally, in general, in the other pHs there was always the presence of a certain proportion of larger structures, both in the absence or presence of JM72, which can be aggregated or even result in dimensional expansion of the bioparticles themselves, caused by processes of electrostatic repulsion between charged groups within the polyelectrolytes.

**Figure 2:**
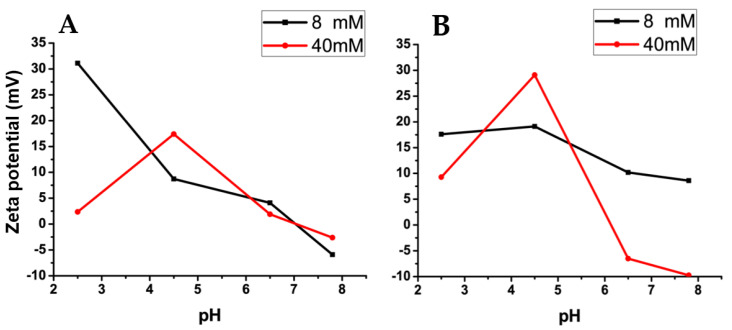
Variation of mean zeta potential as a function of pH in buffers of different concentrations as indicated. (**A**) Results of bioparticles containing JM72. (**B**) Results of bioparticles without JM72. Lines are guide to the eye.

Further comparing the zeta potential results of bioparticles with and without JM72 in the two conditions (Figure 2), a small dependence with pH is evidenced for bioparticles with JM72 (Figure 2A) in the low ionic concentration condition. However, at a high ionic concentration, this dependence practically disappears, except at pH 4.5. Comparing to the blank bioparticle (Figure 2B), pH dependence was lower for the low concentration and increased for the high ionic concentration. Once again, at pH 4.5, there was a more pronounced increase in 40 mM of buffer, which indicates that at this pH and at this concentration, high protonation of chitosan amino groups, concomitant with ionization of alginate, results in a higher zeta potential, equivalently for pure bioparticle and for that containing JM72.

In general, comparing Figure 2A,B, zeta potential results show a similar tendency for bioparticles with and without JM72. The same could be observed in the evaluation of hydrodynamic diameters. Therefore, the presence of JM72 in the bioparticle did not cause an important structural change, suggesting that its inclusion in the composition of the material may be advantageous to enable its application, as it was previously described for polyelectrolytes particles containing hydrophobic drugs [25,26,27,28]. Moreover, considering the pH range in which the bioparticles were evaluated, relaying the pH variation in gastrointestinal tract, it can be inferred that the compact structure of JM72-bioparticle in pH 2.5 and high ionic concentration (Table 5) may facilitate fast transit in stomach, while significant size increase or aggregation, especially in pHs 6.5 and 7.8, can favor mucosal retention in intestine. Hence, the pH-sensitive characteristic of the bioparticle may promote prolonged delivery of the antibacterial with the occurrence of longstanding retention, where the interaction with intestinal epithelial membranes can be favored. Additionally, it has been shown that mucoadhesive polymers as chitosan and penetration enhancers as arginine and tryptophan can actuate in opening of tight junctions and thus trigger bioactives delivery through epithelial cells by temporarily increasing the permeability [42,43,44]. Thus, the presence of these components in the structure of the bioparticle may enhance effective delivery of the antimicrobial as a result of interaction with epithelial membranes and glycoproteins, such as sialic acid [45,46].

### 3.3. Bioparticle Interaction with Model Membrane

Giant liposomes, also known as giant unilamellar vesicles (GUVs), were applied in the present study as a model membrane in order to evaluate the interaction of the bioparticles with a simplified cell membrane made only with lipids. The GUVs advantage is that the micrometer dimension allows direct microscopic observation of the membrane dynamic properties through optical microscopy under controlled parameters. Hence, the changes caused by the variation of physical-chemical conditions of the medium and the interaction with different molecules or structures can be studied, since the behavior of the vesicle membrane can be monitored.

**Figure 3:**
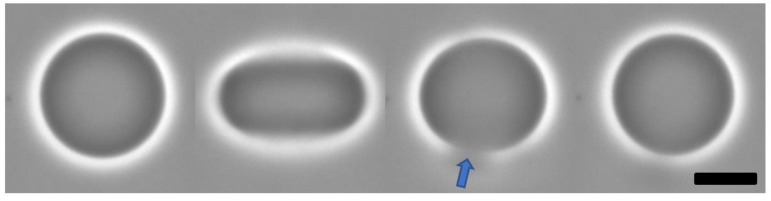
Phase contrast microscopy snapshots showing sequence of electroporation of POPC GUVs in polypeptide–bioparticle solution with deformation produced by electric pulse, pore formation indicated by arrow and final recovery of the spherical shape. All process occurs in less than 100 ms. Bar expands 20 μm for all.

In electroporation of GUVs, the same were in a special electric field chamber, containing saline solution and also dispersed in aqueous solutions containing different concentrations of polypeptide bioparticles. The electric pulses lead to deformation of the vesicles and opening of pore on the membrane. The edge tension of the pores was measured for each individual monitored vesicle. Figure 3 shows a sequence of events during electroporation, evidencing the formation of a large pore on the GUV membrane during milliseconds lifetime. The evolution of the pore closure after the end of the electric pulse could be followed with a fast camera. Afterwards, the GUV recovers the initial shape.

**Figure 4:**
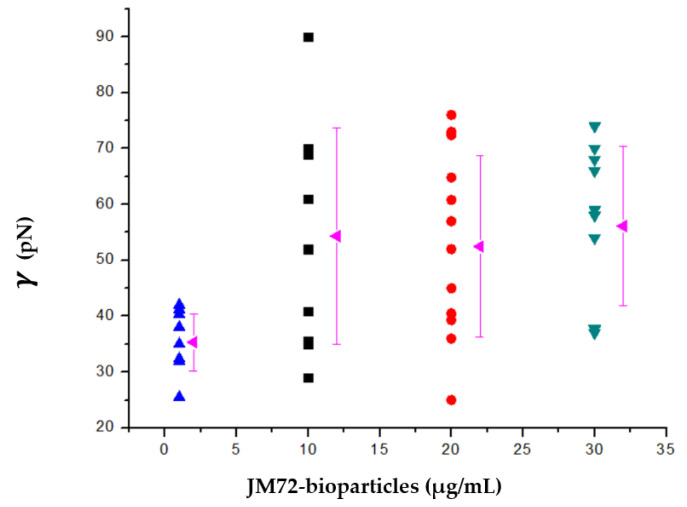
Pore edge tension of POPC giant unilamellar vesicles as a function of JM72-bioparticles concentration. Each point corresponds to a result for a single vesicle and rose triangles correspond to average and variation bars to standard deviation.

In determining the pore edge tension, the stage of pore closing is monitored by extracting the time dependence of the pore size, as previously described [37,47]. The results are shown in Figure 4. For POPC GUVs in solution without bioparticles we found an average edge tension of 35 pN, with a standard deviation of 5 pN. However, for POPC GUVs in a solution with small concentrations of bioparticle, the average edge tension increased to 54 pN at 1 and 2 μg/mL of JM72-bioparticles and to 56 pN at 3 μg/mL of JM72-bioparticles. The large standard deviation, over 10 pN, is inherent to the experimental conditions, since bioparticles are actually dispersed in the GUVs solution and not directly bonded to the lipid membrane, which represents a highly dynamic condition. Despite this, in performing ANOVA statistics (Bonferroni test), a significant increase of average edge tension (*p* < 0.05) in the presence of small concentrations of bioparticles is evidenced, compared to the result in absence of bioparticles.

It is apparent that in the presence of bioparticles, more energy is required to rearrange POPC lipids along the pore walls, leading to edge tension increase, similar to the effect provided by increasing concentrations of cholesterol in GUVs membranes [36]. In our study, the polysaccharides and polypeptide which compose the bioparticle structure may interact with the lipid membrane on the surface of GUVs. Indeed, positive and negative charges of amino, guanidinium and carboxyl groups on the bioparticle plus choline and phosphate on POPC polar head may provide some electrostatic interactions leading to coordination or attachment of bioparticles over the GUVs membrane. Previously, the coordination of free chitosan in solution on DOPC GUVs was described to increase the bending rigidity of the membrane [48]. Recently, thermodynamic interaction between chitosan-alginate nanoparticles and POPG liposomes was described with heat release and significant enthalpic variation [26]. The former and present studies corroborate that the JM72-bioparticles indeed interact with the lipids membrane, leading to important physical changes as a result of effective interaction, which in the case of bioparticles carrying the antimicrobial polypeptide may have implications in effectiveness of antibacterial action.

### 3.4. Antibacterial Activity

**Figure 5:**
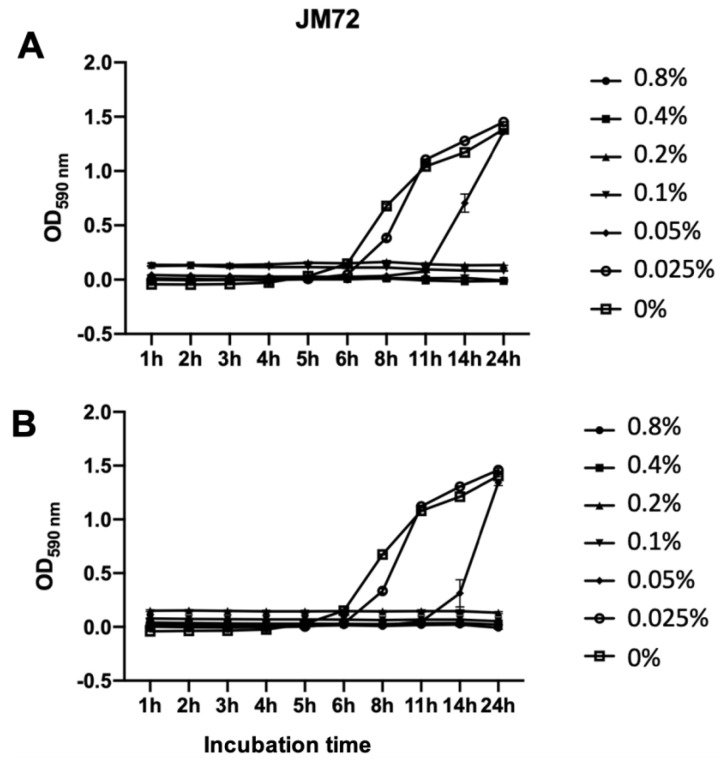
Growth kinetics of *Aeromonas dhakensis* incubated with various concentrations of pure JM72 polypeptide (**A**) and the same carried in bioparticles (**B**). Relative bacterial growth was determined during 24 h using turbidity readings (OD590 nm). Data are shown as mean ± standard deviation from three replicates. Lines are guide to the eye.

The antibacterial activity of polypeptide JM72 alone and complexed to the bioparticle (15 µM JM72 sample) was in vitro evaluated against *Aeromonas dhakensis* isolated from diseased pacu fish (*Piaractus mesopotamicus*) [39]. Figure 5 shows similar results for both conditions, with complete bacterial growth inhibition provided at 0.1%.

In both independent experiments, the control (0%) and the lower concentration of polypeptide (0.025%) showed the same results with a log phase at 6 and 8 h, respectively. At 0.05%, a slight difference was noticeable with a log phase at 11 h for the pure JM72, but an increase to 14 h for JM72-bioparticles. This result may suggest a slight improvement of the antibacterial action of the relatively low concentration of polypeptide when the same is transported by the bioparticle, corroborating that the effective interaction of the bioparticles with cell membranes may improve the effective action of carried bioactives, as previously described for similar praziquantel and invermectin drug release nanoparticles [25,26,27,28].

In the presence of 0.1% (and above) of free or bioparticle-carried JM72, no visible growth of *Aeromonas dhakensis* colonies was detected during 24 h exposure. Thereby, it is evidenced that the antibacterial action of JM72 is effective and that the complexation of the polypeptide with the bioparticles is not inhibiting the antibacterial effect. The unveiled results further evidence that association of the polypeptide with chitosan-*N*-arginine and alginate biopolymers can be advantageous as an antimicrobial delivery system.

The *Aeromonas dhakensis* represents a bacteria with multifactorial pathogenicity [33] and a number of virulence factors, such as a secretion system, toxins and a quorum sensing system, have been described [49,50,51]. Strains of the bacteria have been reported to possess cytotoxic activities in human blood cell lines [52], cause bacteremia [53] and gastroenteritis [54]. As a bacteria mainly found in water [34], the development of JM72-bioparticle as a provider of effective bacterial inhibition in aqueous media represents a promising strategy to fight this pathogenic menace.

## 4. Conclusions

The complexation between biopolymers and JM72 polypeptide led to large negative variation in enthalpy, Gibbs free energy and entropy, providing the production of biopolymers-polypeptide bioparticles with thermodynamic favored conditions. In re-dispersing the freeze-dried particles in aqueous conditions of different ionic strength and various pHs, the bioparticle structural dependence on the solution conditions was roughly maintained with the presence of JM72, hence its inclusion in the bioparticle preserves the bioparticle colloidal characteristics. Moreover, the physical change of increase of pore edge tension on the lipid bilayer of giant vesicles evidenced effective interaction of JM72-bioparticles with the simplified model membrane. Finally, the in vitro antibacterial activity on *Aeromonas dhakensis* of the polypeptide complexed with biopolymers highlights perspectives of the composite bioparticle as an efficient antibacterial material for biomedical applications.

## Figures and Tables

**Table 1:** Thermodynamic parameters obtained for the interaction between alginate and CHarg without polypeptide and in the presence of different concentrations of JM72.

Bioparticle	*N*	*K* (10^10^ M^−1^)	ΔH (kcal/mol)	ΔG (kcal/mol)	TΔS (kcal/mol)
CHargAlg	0.6290 ± 0.0050	0.029 ± 0.004	−725 ± 8.9	−13.93	−711
CHargAlg JM72 (2.5 µM)	0.0368 ± 0.0002	2.95 ± 0.42	−1125 ± 67	−16.65	−1108
CHargAlg JM72 (7.5 µM)	0.0225 ± 0.0007	5.53 ± 0.29	−1369 ± 134	−17.02	−1352
CHargAlg JM72 (15 µM)	0.0147 ± 0.0003	4.17 ± 0.56	−2514 ± 86	−16.86	−2497

**Table 2:** Bioparticle structural characteristics in different buffer solutions, all at 8 mM.

Buffer (pH)	Zeta Potential (mV)	Hydrodynamic Size (nm)	PDI
Citrate 2.5	17.6 ± 8.8	361 ± 60 (100%)	0.52
Acetate 4.5	19.1 ± 6.4	4139 ± 515 (57%)	0.68
		274 ± 26 (43%)	
Phosphate 6.5	10.2 ± 2.9	867 ± 44 (83%)	0.53
		118 ± 18 (17%)	
Phosphate 7.8	8.6 ± 6.4	568 ± 83 (61%)	0.48
		3421 ± 416 (39%)	

**Table 3:** Bioparticle structural characteristics in different buffer solutions, all at 40 mM.

Buffer (pH)	Zeta Potential (mV)	Hydrodynamic Size (nm)	PDI
Citrate 2.5	9.3 ± 6.7	101 ± 18 (100%)	0.21
Acetate 4.5	29.1 ± 9.6	538 ± 228 (62%)	0.73
		5463 ± 523 (30%)	
		94 ± 15 (8%)	
Phosphate 6.5	−6.5 ± 5.6	383 ± 75 (74%)	0.42
		4328 ± 760 (26%)	
Phosphate 7.8	−9.8 ± 6.3	468 ± 91 (92%)	0.43
		2766 ± 728 (8%)	

**Table 4:** JM72-bioparticle structural characteristics in different buffer solutions, all at 8 mM.

Buffer (pH)	Zeta Potential (mV)	Hydrodynamic Size (nm)	PDI
Citrate 2.5	31.1 ± 11.9	856 ± 110 (66%)	0.47
		157 ± 31 (23%)	
		4999 ± 916 (11%)	
Acetate 4.5	8.7 ± 4.5	278 ± 88 (65%)	0.74
		4624 ± 1010 (35%)	
Phosphate 6.5	4.1 ± 7.3	912 ± 206 (89%)	0.51
		1147 ± 305 (11%)	
Phosphate 7.8	−5.9 ± 4.9	203 ± 90 (100%)	0.69

**Table 5:** JM72-bioparticle structural characteristics in different buffer solutions, all at 40 mM.

Buffer (pH)	Zeta Potential (mV)	Hydrodynamic Size (nm)	PDI
Citrate 2.5	2.4 ± 7.3	88 ± 31 (100%)	0.47
Acetate 4.5	17.4 ± 5.5	4799 ± 728 (65%)	0.86
		142 ± 65 (35%)	
Phosphate 6.5	1.9 ± 5.4	334 ± 66 (100%)	0.52
Phosphate 7.8	−2.6 ± 6.1	482 ± 110 (93%)	0.51
		7492 ± 1100 (7%)	

## Data Availability

We declare that our research data are available on reasonable request.

## References

[1] Sun Y., Liu Y., Liu W., Lu C., Wang L. (2015). Chitosan microparticles ionically cross-linked with poly(γ-glutamic acid) as antimicrobial peptides and nitric oxide delivery systems. Biochem. Eng. J..

[2] Gao W., Chen Y., Zhang Y., Zhang Q., Zhang L. (2018). Nanoparticle-based local antimicrobial drug delivery. Adv. Drug. Deliv. Rev..

[3] Gomez A.G., Hosseinidoust Z. (2020). Liposomes for antibiotic encapsulation and delivery. ACS Infect. Dis..

[4] Li D., Fei X., Xu L., Wang Y., Tian J., Li Y. (2022). Pressure-sensitive antibacterial hydrogel dressing for wound monitoring in bed ridden patients. J. Colloid Interface Sci..

[5] Tamahkar E., Bakhshpour M., Denizil A. (2019). Molecularly imprinted composite bacterial cellulose nanofibers for antibiotic release. J. Biomater. Sci..

[6] Nair A., Mallya R., Suvarna V., Khan T.A., Momin M., Omri A. (2022). Nanoparticles—Attractive carriers of antimicrobial essential oils. Antibiotics.

[7] Boczar D., Michalska K. (2022). Cyclodextrin inclusion complexes with antibiotics and antibacterial agents as drug-delivery systems—A pharmaceutical perspective. Pharmaceutics.

[8] Catauro M., Tranquillo E., Poggetto G.D., Naviglio S., Barrino F. (2020). Antibacterial properties of sol–gel biomaterials with different percentages of PEG or PCL. Macromol. Symp..

[9] Jian Y., Chen Y., Song Z., Tan Z., Cheng J. (2021). Recent advances in design of antimicrobial peptides and polypeptides toward clinical translation. Adv. Drug Del. Rev..

[10] Kundu R. (2020). Cationic amphiphilic peptides: Synthetic antimicrobial agents inspired by nature. ChemMedChem.

[11] Cirioni O., Silvestri C., Ghiselli R., Kamysz W., Minardi D., Castelli P., Orlando F., Kamysz E., Provinciali M., Muzzonigro G. (2013). In vitro and in vivo effects of sub-mics of pexiganan and imipenem on *Pseudomonas aeruginosa* adhesion and biofilm development. Le Infez. Med..

[12] Rajchakit U., Sarojini V. (2017). Recent developments in antimicrobial-peptide-conjugated gold nanoparticles. Bioconjugate Chem..

[13] Teixeira M.C., Carbone C., Sousa M.C., Espina M., Garcia M.L., Sanchez-Lopez E., Souto E.B. (2020). Nanomedicines for delivery of antimicrobial peptides. Nanomaterials.

[14] D’Angelo I., Casciaro B., Miro A., Quaglia F., Mangoni M.L., Ungaro F. (2015). Overcoming barriers in *Pseudomonas aeruginosa* lung infections: Engineered nanoparticles for local delivery of a cationic antimicrobial peptide. Coll. Surf. B Biointerfaces.

[15] Ma B., Chen Y., Hu G., Zeng Q., Lv X., Hwan Oh D., Fu X., Jin Y. (2022). Ovotransferrin antibacterial peptide coupling mesoporous silica nanoparticle as an effective antibiotic delivery system for treating bacterial infection in vivo. ACS Biomater. Sci. Eng..

[16] Sonia T.A., Sharma C.P. (2011). Chitosan and its derivatives for drug delivery perspective. Adv. Polym. Sci..

[17] Rodrigues S., Dionisio M., Lopez C.R., Greha A. (2012). Biocompatibility of chitosan carriers with application in drug delivery. J. Funct. Biomater..

[18] Mulinti P., Shreffler J., Hasan R., Dea M., Brooks A.E. (2021). Infection responsive smart delivery of antibiotics using recombinant spider silk nanospheres. Pharmaceutics.

[19] Rinaldi F., Hanieh P., Sennato S., De Santis F., Forte J., Fraziano M., Casciardi S., Marianecci C., Bordi F., Carafa M. (2021). Rifampicin–liposomes for *Mycobacterium abscessus* infection treatment: Intracellular uptake and antibacterial activity evaluation. Pharmaceutics.

[20] Pabon B.Y. (2015). Synthesis of Peptide-Functionalized Chitosan Polymeric Nanoparticles and Evaluation of Their In Vitro Activity against *Escherichia coli* and *Staphylococcus aureus*. Master’s Thesis.

[21] Philibert T., Lee B.H., Fabien N. (2017). Current status and new perspectives on chitin and chitosan as functional biopolymers. Appl. Biochem. Biotechnol..

[22] Collado-Gonzalez M., Esteban M.A. (2022). Chitosan-nanoparticles effects on mucosal immunity: A systematic review. Fish Shellfish Immunol..

[23] Bannikova A., Rasumova L., Evteev A., Evdokimov I., Kasapis S. (2017). Protein-loaded sodium alginate and carboxymethyl cellulose beads for controlled release under simulated gastrointestinal conditions. Food Sci. Technol..

[24] Tonnesen H.H., Karlsen J. (2002). Alginate in drug delivery systems. Drug Dev. Ind. Pharm..

[25] Patta A.C.M.F., Mathews P.D., Madrid R.R.M., Rigoni V.L.S., Silva E.R., Mertins O. (2020). Polyionic complexes of chitosan-*N*- arginine with alginate as pH responsive and mucoadhesive particles for oral drug delivery applications. Int. J. Biol. Macromol..

[26] Mathews P.D., Patta A.C.M.F., Madrid R.R.M., Ramirez C.A.B., Pimenta B.V., Mertins O. (2021). Efficient treatment of fish intestinal parasites applying membrane-penetrating oral drug delivery nanoparticle. ACS Biomater. Sci. Eng..

[27] Madrid R.R.M., Mathews P.D., Patta A.C.M.F., Gonzales-Flores A.P., Ramirez C.A.B., Rigoni V.L.S., Tavares-Dias M., Mertins O. (2021). Safety of oral administration of high doses of ivermectin by means of biocompatible polyelectrolytes formulation. Heliyon.

[28] Madrid R.R.M., Mertins O., Tavares-Dias M., Flores-Gonzales A.P., Patta A.C.M.F., Ramirez C.A.B., Rigoni V.L.S., Mathews P.D. (2021). High compliance and effective treatment of fish endoparasitic infections with oral drug delivery nanobioparticles: Safety of intestinal tissue and blood parameters. J. Fish Dis..

[29] Silva J.M.D. (2017). Estudos de síntese, conformação e atividade biológica de análogos do peptídeo antimicrobiano longipina. Master’s Thesis.

[30] Wittemann A., Ballauff M. (2006). Interaction of proteins with linear polyelectrolytes and spherical polyelectrolyte brushes in aqueous solution. Phys. Chem. Chem. Phys..

[31] Xu X., Angioletti-Uberti S., Lu Y., Dzubiella J., Ballauff M. (2019). Interaction of proteins with polyelectrolytes: Comparison of theory to experiment. Langmuir.

[32] Kitagawa H., Ohge H., Yu L., Kayama S., Hara T., Kashiyama S., Kajihara T., Hisatsune J., Sueda T., Sugai M. (2020). *Aeromonas dhakensis* is not a rare cause of *Aeromonas* bacteremia in Hiroshima, Japan. J. Infect. Chemother..

[33] Chen P.L., Lamy B., Ko W.C. (2016). *Aeromonas dhakensis*, an increasingly recognized human pathogen. Front. Microbiol..

[34] Esteve C., Alcaide E., Blasco M.D. (2012). *Aeromonas hydrophila* subsp. *dhakensis* isolated from feces, water and fish in Mediterranean Spain. Microbes Environ..

[35] Angelova M.I., Dimitrov D.S. (1986). Liposome electroformation. Faraday Discus. Chem. Soc..

[36] Karatekin E., Sandre O., Guitouni H., Borghi N., Puech P.H., Brochard-Wyart F. (2003). Cascades of transient pores in giant vesicles: Line tension and transport. Biophys. J..

[37] Leomil F.S.C., Zoccoler M., Dimova R., Rike K.A. (2021). PoET: Automated approach for measuring pore edge tension in giant unilamellar vesicles. Bioinform. Adv..

[38] Mathews P.D., Patta A.C.M.F., Gonçalves J.V., Gama G.S., Garcia I.T.S., Mertins O. (2018). Targeted drug delivery and treatment of endoparasites with biocompatible particles of pH-responsive structure. Biomacromolecules.

[39] Carriero M.M., Maia A.A.M., Sousa R.L.M., Henrique-Silva F. (2016). Characterization of a new strain of *Aeromonas dhakensis* isolated from diseased pacu fish (*Piaractus mesopotamicus*) in Brazil. J. Fish Dis..

[40] Rinaudo M. (2006). Chitin and chitosan: Properties and applications. Prog. Polym. Sci..

[41] Schatz C., Viton C., Delair T., Pichot C., Domard A. (2003). Typical physicochemical behaviors of chitosan in aqueous solution. Biomacromolecules.

[42] Sonaje K., Chuang E.-Y., Lin K.-J., Yen T.-C., Su F.-Y., Tseng M.T., Sung H.-W. (2012). Opening of epithelial tight junctions and enhancement of paracellular permeation by chitosan: Microscopic, ultrastructural, and computed-tomographic observations. Mol. Pharm..

[43] Wang J., Kong M., Zhou Z., Yan D., Yu X., Cheng X., Feng C., Liu Y., Chen X. (2017). Mechanism of surface charge triggered intestinal epithelial tight junction opening upon chitosan nanoparticles for insulin oral delivery. Carbohydr. Polym..

[44] Kamei N., Tamiwa H., Miyata M., Haruna Y., Matsumura K., Ogino H., Hirano S., Higashiyama K., Takeda-Morishita M. (2018). Hydrophobic amino acid tryptophan shows promise as a potential absorption enhancer for oral delivery of biopharmaceuticals. Pharmaceutics.

[45] Artursson P., Lindmark T., Davis S.S., Illum L. (2005). Pharmaceutical aspects of polymeric nanoparticles for oral drug delivery. J. Biomed. Nanotech..

[46] Artursson P., Lindmark T., Davis S.S., Illum L. (1994). Effect of chitosan on the permeability of monolayers of intestinal epithelial cells (Caco-2). Pharm. Res..

[47] Portet T., Dimova R. (2010). A New method for measuring edge tensions and stability of lipid bilayers: Effect of membrane composition. Biophys. J..

[48] Mertins O., Dimova R. (2013). Insights on the interactions of chitosan with phospholipid vesicles. Part II: Membrane stiffening and pore formation. Langmuir.

[49] Tomas J.M. (2012). The main *Aeromonas* pathogenic factors. ISRN Microbiol..

[50] Chen P.-L., Wu C.-J., Tsai P.-J., Tang H.-J., Chuang Y.-C., Lee N.-Y., Lee C.-C., Li C.-W., Li M.-C., Chen C.-C. (2014). Virulence diversity among bacteremic *Aeromonas* isolates: Ex vivo, animal, and clinical evidences. PLoS ONE.

[51] Mosser T., Talagrand-Reboul E., Colston S.M., Graf J., Figueras M.J., Jumas-Bilak E., Lamy B. (2015). Exposure to pairs of *Aeromonas* strains enhances virulence in the *Caenorhabditis elegans* infection model. Front. Microbiol..

[52] Morinaga Y., Yanagihara K., Araki N., Harada Y., Yamada K., Akamatsu N., Matsuda J., Nishino T., Hasegawa H., Izumikawa K. (2011). Clinical characteristics of seven patients with *Aeromonas* septicemia in a Japanese hospital. Tohoku J. Exp. Med..

[53] Wu C.-J., Hsuan-Chen W., Hsueh P.-R., Chang M.-C., Tsai P.-J., Shih H.-I., Wang H.-C., Chou P.-H., Ko W.-C. (2015). Clinical implications of species identification in monomicrobial *Aeromonas* bacteremia. PLoS ONE.

[54] Wu C.-J., Tsai P.-J., Chen P.-L., Wu I.-C., Lin Y.-T., Chen Y.-H., Wang L.-R., Ko W.-C. (2012). *Aeromonas aquariorum* septicemia and enterocolitis in a cirrhotic patient. Diagn. Microbiol. Infect. Dis..

